# Patient-Reported Outcomes and Functional Recovery After Treatment for Laryngeal Cancer: A Scoping Review of Instruments, Domains, and Clinical Integration

**DOI:** 10.3390/jcm15134872

**Published:** 2026-06-23

**Authors:** Ion Costel Epuraș, Alexandru Florian Crișan, Nicolae Constantin Balica, Cristian Ion Moț, Adrian Mihail Sitaru, Mihaela Iuliana Sîrbu, Andreea Mihaela Banta, Dan Iovanescu, Carina Gib, Gheorghe Iovanescu

**Affiliations:** 1 Doctoral School, “Victor Babes” University of Medicine and Pharmacy Timisoara, Eftimie Murgu Square 2, 300041 Timisoara, Romania; ion.epuras@umft.ro (I.C.E.); mihaela.sirbu@umft.ro (M.I.S.); andreea.banta@umft.ro (A.M.B.); dan.iovanescu@umft.ro (D.I.); carina.gib@umft.ro (C.G.); 2 Oftalmo Sensory-Tumor Research Center-ORL (EYE-ENT), “Victor Babes” University of Medicine and Pharmacy Timisoara, Eftimie Murgu Sq. No. 2, 300041 Timisoara, Romaniaion.mot@umft.ro (C.I.M.); giovanescu@umft.ro (G.I.); 3 Research Center for the Assessment of Human Motion, Functionality and Disability (CEMFD), “Victor Babes” University of Medicine and Pharmacy Timisoara, Eftimie Murgu Square 2, 300041 Timisoara, Romania; 4 Pulmonary Rehabilitation Center, Clinical Hospital of Infectious Diseases and Pulmonology, “Victor Babes” University of Medicine and Pharmacy Timisoara, Gheorghe Adam Street 13, 300310 Timisoara, Romania; 5 Department IX, Discipline of Otolaryngology, “Victor Babes” University of Medicine and Pharmacy Timisoara, Eftimie Murgu Square 2, 300041 Timisoara, Romania; 6 Department of Pediatric Surgery, “Louis Turcanu” Emergency Clinical Hospital for Children, Iosif Nemoianu Street 2, 300011 Timisoara, Romania; adrian.sitaru@umft.ro

**Keywords:** laryngeal cancer, patient-reported outcomes, functional recovery

## Abstract

**Background/Objectives**: Treatment for laryngeal cancer often impacts voice, swallowing, communication, and quality of life. Patient-reported outcome measures (PROMs) are increasingly used to evaluate survivorship, but their application and connection with objective functional measures vary widely. The objective was to explore how PROMs are used in laryngeal cancer research, identify the functional areas they assess, analyze their link with objective clinical outcomes, and identify methodological gaps in current studies. **Methods**: This scoping review followed PRISMA-ScR guidelines. Searches were conducted in PubMed/MEDLINE, Scopus, and Web of Science from their start until April 2026. Included studies involved adults with laryngeal cancer reporting PROMs and/or objective functional outcomes. Data on study features, PROM tools, evaluated domains, and how PROMs relate to objective outcomes were extracted and summarized descriptively. **Results**: Ninety-five studies with 10,807 participants were included. Most were observational (84.2%) and conducted at a single center (72.6%). Voice-related outcomes were the most common (86.3%), followed by psychological (72.6%) and swallowing outcomes (65.3%). Less frequently assessed were nutritional (22.1%) and supportive care domains (41.1%). The Voice Handicap Index family was the most used PROM group (30.5%). Over half the studies reported PROMs and objective measures separately without statistical integration (51.6%), while only 13.7% performed analytical integration, and none used predictive multivariable models. Significant variation existed in PROM choices, assessed domains, and integration approaches. **Conclusions:** PROM use in laryngeal cancer survivorship research is heterogeneous and predominantly focused on voice-related outcomes. Limited analytical integration with objective measures hampers a comprehensive understanding of recovery. There is a need for standardized, multidimensional assessment frameworks that include functional, nutritional, psychosocial, and objective outcomes to effectively support patient-centered survivorship care and rehabilitation planning.

## 1. Introduction

Laryngeal cancer is a distinct subgroup of head and neck cancers that significantly affects survival and crucial functions such as voice, swallowing, and airway protection [1]. Although progress in surgical and oncological treatments has enhanced the overall management of laryngeal cancer, survival outcomes remain variable, and treatments often cause significant functional impairments such as dysphonia, swallowing issues, and laryngeal dysfunction, which greatly affect quality of life [1]. Specifically, total laryngectomy results in the permanent loss of natural voice and significant changes in communication, which can have major psychosocial and social impacts [2]. Since successful treatment of laryngeal cancer involves not only oncologic control but also the maintenance of function and overall well-being, evaluating patient-centered outcomes has gained increasing importance.

Patient-reported outcome measures (PROMs) have become vital tools for assessing treatment results, emphasizing the importance of patient-centered care [3,4]. Unlike solely clinician-reported or physiological measures, PROMs reflect patients’ perceptions of symptoms, functional limitations, and health-related quality of life, providing additional insight into disease burden and recovery [5,6]. In laryngeal cancer, where treatments can significantly impact speech, swallowing, and social engagement, PROMs are especially important for guiding research and clinical choices [7,8].

However, the use of PROMs and related functional assessments in this field remains heterogeneous, with studies employing different tools, timing, study designs, and methods for linking patient-reported outcomes to clinical outcomes [4,9,10,11,12,13,14,15]. This variability makes it harder to compare studies and complicates evidence synthesis.

Besides methodological differences, outcome reporting in laryngeal cancer tends to be unbalanced. Previous studies have frequently emphasized voice-related outcomes, whereas nutritional status, psychological well-being, and supportive care needs are less consistently assessed [16,17,18,19]. Studies also differ significantly in whether they combine general quality-of-life tools, domain-specific PROMs, and objective functional measures like voice or swallowing assessments [12,13,14,15,17]. Although PROMs and objective outcomes are often reported within the same studies, they are commonly presented in parallel rather than integrated analytically through correlation analyses, regression models, or predictive frameworks, limiting clinical interpretability and understanding of how patient perceptions relate to measurable functional recovery [13,17,19,20,21,22].

To our knowledge, no scoping review has comprehensively mapped the range of PROMs used in laryngeal cancer, the domains assessed, and their integration with objective clinical outcomes. Given the diversity of instruments, outcome domains, and approaches to integrating PROMs with objective measures, a scoping review is appropriate for mapping the existing evidence, identifying patterns in outcome assessment, and highlighting methodological gaps in the field.

This scoping review aimed to systematically map the use of patient-reported outcome measures in laryngeal cancer, identify the domains evaluated, analyze how PROMs are integrated with objective clinical outcomes, and highlight methodological gaps to inform future research and aid in developing more comprehensive outcome assessment frameworks.

## 2. Materials and Methods

### 2.1. Protocol and Registration

This scoping review was carried out and documented in accordance with the PRISMA Extension for Scoping Reviews (PRISMA-ScR) guidelines [23]. The protocol was developed beforehand and registered on the Open Science Framework (OSF) at https://osf.io/4au92 (accessed on 18 May 2026).

### 2.2. Eligibility Criteria

Studies were included if they met the following conditions: (1) involved adult patients diagnosed with laryngeal cancer; (2) reported patient-reported outcome measures (PROMs) related to quality of life or functional outcomes; and/or (3) contained objective clinical outcomes such as voice assessments, swallowing evaluations, nutritional status, or other functional metrics. Quantitative observational and interventional primary studies were considered eligible.

Studies were excluded if they (1) did not focus on laryngeal cancer as the primary population; (2) were review articles, case reports, editorials, conference abstracts without full text, or non-peer-reviewed publications; or (3) had unavailable full texts despite reasonable retrieval attempts. There were no restrictions regarding the year of publication. Only studies published in English were included, in accordance with the eligibility criteria outlined in the prospectively registered review protocol. Although machine-assisted translation has improved, reducing language barriers in evidence synthesis, limiting inclusion to English-language publications is intended to maintain consistency in study selection, data extraction, and interpretation throughout the review.

### 2.3. Search Strategy

A comprehensive search strategy was developed using a combination of Medical Subject Headings (MeSH) and free-text keywords related to laryngeal cancer and patient-reported outcomes. Three main concept groups were combined using Boolean operators (AND/OR): (1) laryngeal cancer terms (“laryngeal cancer”, “larynx cancer”, “glottic cancer”, “supraglottic cancer”); (2) patient-reported outcome concepts (“patient-reported outcome”, “PROM”, “PROMs”, “quality of life”, “QoL”, “health-related quality of life”, “HRQoL”); and (3) functional outcome concepts (“voice”, “voice outcome”, “voice quality”, “speech”, “swallowing”, “dysphagia”, “functional outcome”, and “functional recovery”). Searches were conducted in PubMed/MEDLINE, Scopus, and Web of Science from database inception to April 2026. The search syntax was adapted for each database. Complete database-specific search strategies are provided in Supplementary File S1.

### 2.4. Selection of Sources of Evidence

**Figure 1:**
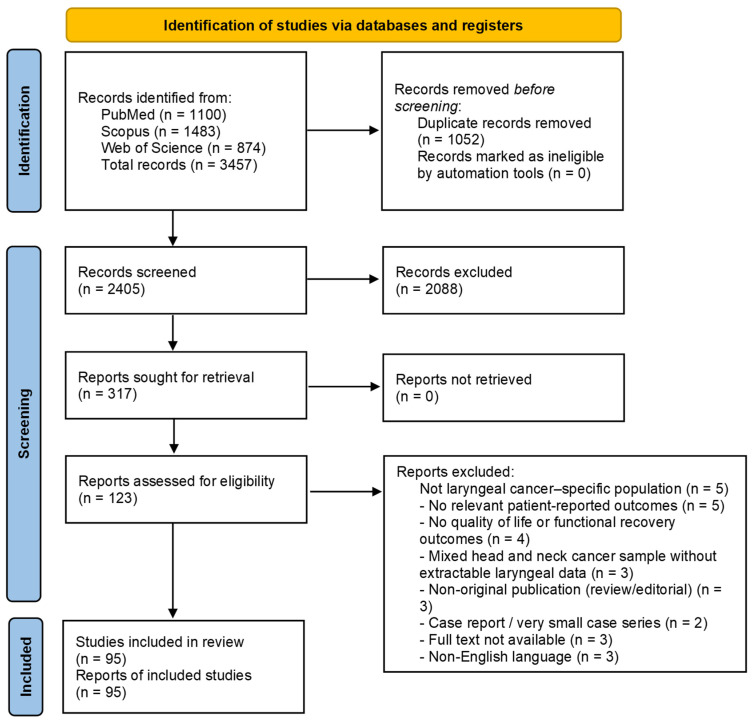
PRISMA flow diagram.

All retrieved records were imported into Rayyan (Qatar Computing Research Instatute, Hamad Bin Khalifa University, Doha, Qatar) for screening and management. After removing duplicates, two reviewers independently screened titles and abstracts based on predefined eligibility criteria. Full texts of potentially relevant studies were then assessed for inclusion. Any disagreements were resolved through discussion and consensus. The study selection process is presented in a PRISMA-ScR flow diagram (Figure 1). A PRISMA-ScR checklist is provided in Supplementary File S2.

Data were charted using a standardized, pilot-tested charting form created in Microsoft Excel. This form contained predefined variables covering study characteristics, PROM instruments, assessed domains, objective outcomes, supportive care factors, and the integration patterns of PROMs with clinical outcomes. The recorded data were then reviewed and verified to ensure accuracy and consistency.

### 2.5. Data Items

The extracted data from each study included publication details (such as the first author, year, journal), geographic location, study design and setting, sample size, patient characteristics, treatment type, PROM instruments used, assessed domains (e.g., voice, swallowing, psychological, nutritional, supportive care), objective clinical outcomes, and follow-up period.

Additionally, the level of integration between PROMs and objective clinical outcomes was categorized using an a priori framework developed by the review team for this scoping review to characterize the degree of analytical connection between patient-reported and objective measures. The framework comprised four levels: Level 0 (PROMs only), Level 1 (parallel reporting without statistical analysis), Level 2 (analytical integration, such as correlation or association analyses), and Level 3 (predictive integration using multivariable models). This framework was used as a conceptual mapping tool to support descriptive synthesis rather than as a formal, validated rating instrument.

A formal risk-of-bias assessment was not conducted because the main goal of this scoping review was to map existing evidence rather than assess study quality. However, to improve interpretability, a supplementary methodological overview summarizing study designs, settings, and follow-up characteristics of the included studies is included (Supplementary Table S1).

### 2.6. Results Synthesis

The data collected were combined through descriptive and analytical methods. Study features, PROM application, and domains were summarized with frequencies and percentages. The use and reporting of PROM instruments by domain, along with supportive care variables, were analyzed to find patterns and inconsistencies.

The review team initially created the integration framework specifically for this scoping review. Its purpose was driven by the noticeable variation in how studies reported and analyzed patient-reported outcome measures (PROMs) and objective clinical outcomes. The framework serves as a practical evidence-mapping tool that classifies the degree of analytical integration between patient-reported and objective outcomes, ranging from PROM-only reports (Level 0) to predictive multivariable analyses (Level 3). These categories were established prior to data extraction and were applied uniformly throughout the review. The framework aims to facilitate descriptive synthesis and evidence mapping, rather than act as a validated method for assessment.

**Figure 2:**
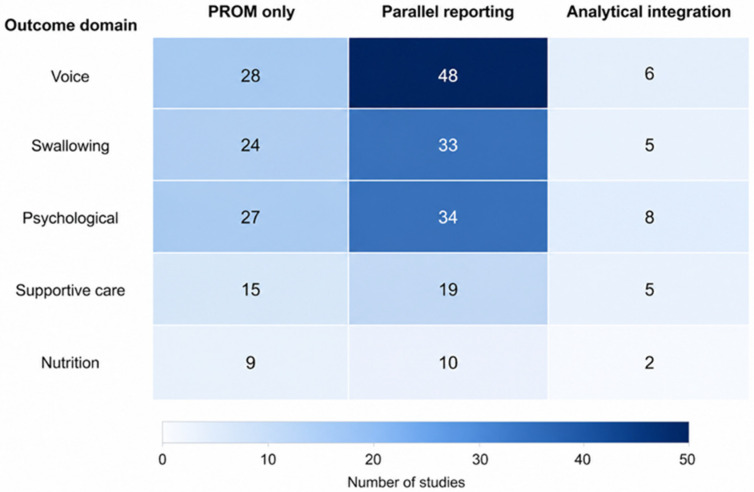
Heatmap of outcome domains and levels of integration between patient-reported and objective clinical outcomes. Values represent the number of studies contributing to each domain–integration category combination. Outcome domains were identified during data extraction and cross-tabulated with the predefined integration levels. Individual studies could contribute to multiple outcome domains when more than one PROM category was assessed; therefore, values are not mutually exclusive study counts. Darker shades indicate greater representation of studies within each domain–integration category combination.

For visualization, outcome domains identified during data extraction—such as voice, swallowing, psychological, supportive care, and nutrition—were cross-tabulated with the predefined integration levels. The resulting frequency counts were used to create the heatmap shown in Figure 2. Studies evaluating multiple outcome domains were included in each relevant domain, allowing them to contribute to more than one domain–integration category.

Findings were presented in tables and accompanied by narrative synthesis to emphasize main trends, methodological differences, and gaps in the existing literature.

### 2.7. Use of Generative Artificial Intelligence

Generative artificial intelligence tools were used to support language refinement, text structuring, and drafting during manuscript preparation. All screening decisions, data extraction, interpretation of findings, and final manuscript revisions were performed and verified by the authors.

## 3. Results

### Selection of Evidence Sources

A total of 3457 records were identified through database searches (PubMed/MEDLINE *n* = 1100, Scopus *n* = 1483, Web of Science *n* = 874). After the removal of 1052 duplicate records, 2405 records remained for title and abstract screening. Of these, 2088 records were excluded for not meeting the eligibility criteria, leaving 317 records for further assessment. Following additional screening, 123 full-text articles were assessed for eligibility. Of these, 28 were excluded, resulting in 95 studies included in the final scoping review (see Figure 1).

The key features of the included studies are summarized in Table 1. The included studies were published from 2001 to 2025, with a median publication year of 2016 (IQR 2012–2021). The majority came from Europe (58.9%) and Asia (24.2%), and most were observational (84.2%) and single-center studies (72.6%). Cross-sectional and prospective designs were the most common, each accounting for 27.4%. The median sample size was 69 participants (IQR 43.5–95.5), and follow-up data were available in 83.2% of the studies.

A detailed study-level methodological overview, including study design, patient population, treatment type, PROMs used, sample size, and follow-up characteristics, is provided in Supplementary Table S1.

Table 2 outlines the patient-reported outcome measures (PROMs) employed in the included studies. Voice-related tools were the most common, with the Voice Handicap Index (VHI) family being the most frequently used PROM group at 45.3%. Instruments assessing general and head-and-neck–specific quality of life, such as the EORTC QLQ-C30 and EORTC QLQ-H&N35, were also widely adopted, each appearing in nearly a third of the studies. Swallowing-specific assessments such as the M. D. Anderson Dysphagia Inventory (MDADI) and SWAL-QOL were used less frequently. Meanwhile, communication and psychosocial-focused measures, including the S-SECEL family, exhibited more variability. Several other PROMs were identified but only sporadically employed, highlighting the considerable heterogeneity in outcome assessment methods across the literature.

Table 3 provides an overview of patient-reported outcome measures and the distribution of their assessed domains across the included studies. Figure 2 shows how these domains are integrated with objective clinical outcomes. While PROMs were widely used, the focus across domains was uneven. There was a strong emphasis on voice-related outcomes, whereas supportive care and nutritional areas were notably underrepresented. As depicted in Figure 2, most studies across all domains reported results in parallel, with fewer instances of analytical integration.

Because individual studies frequently assessed multiple outcome domains, a single study could contribute to more than one domain–integration category. Consequently, the values shown represent domain-specific study counts rather than mutually exclusive study categories. The majority of studies were concentrated at the level of parallel reporting, while analytical integration was relatively rare across all domains.

Despite widespread PROM use, analytical integration with objective outcomes was uncommon. As shown in Table 4, most studies (51.6%) reported PROMs and objective outcomes simultaneously but did not perform statistical analyses, referred to as Level 1 integration. A considerable portion (34.7%) relied solely on PROMs (Level 0), while only 13.7% carried out analytical integration methods like correlation or group comparisons (Level 2). Notably, no study demonstrated Level 3 predictive integration.

## 4. Discussion

This scoping review highlights four main findings. First, while patient-reported outcome measures (PROMs) are commonly used in laryngeal cancer research, their use is fragmented, with significant variation in the instruments used and little standardization across studies. Second, the assessment of outcomes mainly focuses on voice-related measures, with nutritional support, care, and psychosocial aspects being less frequently addressed. Third, although many studies report both PROMs and objective functional outcomes, most present these separately rather than integrating them analytically, with few achieving higher-level integration and none employing predictive models. Finally, the evidence mainly comes from observational, single-center studies, exposing current research’s methodological limitations. Overall, these findings indicate that patient-centered outcome assessment has grown in importance in laryngeal cancer research, but key gaps still exist in comprehensiveness, standardization, and clinical use.

### 4.1. Heterogeneity and Fragmentation of PROM Instruments

This review revealed significant variability in how PROMs are selected and used in laryngeal cancer research. While certain instruments, such as the VHI family, EORTC questionnaires, SECEL adaptations, MDADI, SF-36, and UW-QOL, were frequently employed, their application was inconsistent across different studies. The choice of PROMs was heavily influenced by factors like treatment type, functional focus, and specific research goals. For example, studies on early glottic cancer, cordectomy, radiotherapy, or partial laryngectomy tended to focus on voice-related outcomes [17,24,25,26,27]. In contrast, research involving total laryngectomy or rehabilitation after laryngectomy more often prioritized measures related to communication, psychosocial adjustment, and social reintegration [20,28,29].

Voice-specific PROMs were the most consistently used patient-reported tools across the included studies. Research assessing transoral laser microsurgery, cordectomy, and conservative laryngeal surgery often combined VHI with objective voice assessments like acoustic analysis, maximum phonation time, GRBAS ratings, videolaryngostroboscopy, or multidimensional perceptual evaluation protocols [17,24,25,26,30,31,32,33,34]. This multidimensional approach shows that relying solely on objective voice measures might not fully capture the patient’s subjective burden of dysphonia after laryngeal cancer treatment. Multiple studies have shown that patients perceive ongoing voice impairments despite good oncologic or functional outcomes, highlighting the need to include patient-reported voice handicap in survivorship evaluations [17,24,25,26].

Studies involving radiotherapy showed significant variability in PROM combinations and assessment methods. Some studies mainly examined communication experiences using S-SECEL combined with EORTC tools [30,31,32,33,34], whereas multidimensional assessment protocols frequently incorporated perceptual, acoustic, and aerodynamic voice analyses alongside patient-reported outcomes [31,32,33]. Longitudinal studies also showed that, even as many functional and quality-of-life aspects improved over time, communication and voice-related impairments often persisted beyond the initial treatment phase [30,31,32,33,34].

In contrast, studies involving total laryngectomy more often evaluated psychosocial adjustment, communication reintegration, and social functioning [20,28,29]. SECEL-based instruments played an important role in this context, as they evaluated communication avoidance, challenges with environmental speech, changes in self-perception, and social participation following the loss of natural voice [20,28,29]. Studies involving total laryngectomy more often focus on communication reintegration, psychosocial adjustment, and social functioning. Communication-centered PROMs reveal the significant effect of voice loss on social participation, self-view, and quality of life. Overall, the data indicate that different treatment methods steer the focus of survivorship assessments: organ-preservation approaches tend to highlight voice-related outcomes, while total laryngectomy research emphasizes communication adaptation and psychosocial recovery [20,28].

The presence of both generic quality-of-life questionnaires and domain-specific PROMs highlights an important methodological point in this review. Instruments like EORTC QLQ-C30, EORTC QLQ-H&N35, SF-36, UW-QOL, and WHOQOL-B facilitate a comprehensive assessment of physical, emotional, and social well-being [20,27,28,29,30,31,32,33,34,35,36]. Instruments like VHI, SECEL, and MDADI offered a more detailed evaluation of specific survivorship areas, such as dysphonia, communication difficulties, and dysphagia [17,24,25,26,27]. Several studies deliberately combined these methods, showing moderate links between communication dysfunction and overall quality-of-life scores, while also indicating that these tools measure partly separate constructs [30,31,32,33,34].

The variation observed indicates a lack of a standardized core outcome framework in this research area. Present studies often select PROMs based on the primary functional outcome of the treatment, such as voice preservation following cordectomy or communication adaptability following total laryngectomy.

### 4.2. Imbalance of Functional Domains

The current review also identified a notable imbalance in the domains evaluated across the included studies. Voice-related outcomes were the most assessed, appearing in 86.3% of studies, while nutrition-related outcomes appeared in only 22.1%, and supportive care outcomes in 41.1%. This dominance of voice assessment is understandable from a clinical perspective, as phonation is one of the most visible and functionally significant effects of laryngeal cancer treatment. Studies focusing on early glottic cancer, cordectomy, radiotherapy, and larynx-preserving surgery consistently emphasized voice handicap, acoustic measures, perceptual voice quality, and communication outcomes [13,17,25,37,38].

Nevertheless, functional recovery after laryngeal cancer is multidimensional, but current literature primarily focuses on voice. Swallowing dysfunction is a prime example of an underrepresented aspect of survivorship. Several studies have shown ongoing issues such as dysphagia, aspiration symptoms, social eating challenges, dry mouth, dependence on feeding tubes, use of nutritional supplements, and weight loss following both surgical and nonsurgical treatments [13,25,37,38,39,40]. Importantly, anatomical preservation does not necessarily imply functional preservation. Several studies suggest that anatomical preservation does not necessarily translate into superior swallowing outcomes, as clinically relevant dysphagia and nutritional compromise may persist across both surgical and organ-preservation treatment strategies [40]. Similarly, research utilizing SWAL-QOL, MDADI, EORTC QLQ-H&N35, and FACT-H&N showed that swallowing difficulties significantly impact social life, confidence in eating, and overall quality of life [13,17,37,38,39].

The limited use of swallowing-specific PROMs is notable, especially amid the increasing focus on multidisciplinary care for head and neck cancer survivors. Modern treatment often involves collaboration among otolaryngologists, speech and language therapists, dietitians, oncologists, and rehabilitation experts. Early swallowing evaluations, specialized rehabilitation, dietary advice, and long-term follow-up can help reduce persistent swallowing issues and improve patient-reported outcomes. This review indicates that swallowing-related PROMs are still underused despite their clinical importance, which may hinder thorough assessment of rehab success and functional recovery.

Nutritional consequences were studied less systematically, despite their clear clinical significance. Dysphagia, aspiration risk, dry mouth, sticky saliva, altered taste, decreased oral intake, feeding difficulties, and chronic weight loss can significantly lead to malnutrition, hindered functional recovery, decreased physical reserves, and lower long-term quality of life in laryngeal cancer survivors [14,41,42,43,44,45]. Multiple studies have found ongoing swallowing and social eating challenges after total laryngectomy or chemoradiotherapy. Additionally, reliance on tube feeding and nutritional supplements was linked to lower quality-of-life outcomes [41,42,43,44,45]. In the reviewed literature, ongoing nutritional issues, such as appetite loss, feeding difficulties, dry mouth, altered taste, dependence on tube feeding, and dietary restrictions, were consistently linked to lower quality-of-life outcomes. These results indicate that nutritional health is a crucial yet often under-evaluated aspect of survivorship following laryngeal cancer treatment [42,43].

However, most studies treat nutrition-related issues as isolated questionnaire items, like supplement use, appetite loss, feeding tube dependence, weight loss, swallowing problems, or social eating limitations. They often do not comprehensively assess nutritional health using dedicated assessment tools. This shows that nutritional health remains poorly integrated into PROM-based assessments, despite its important links to swallowing function, treatment toxicity, rehabilitation potential, and overall survivorship.

Psychosocial functioning has become an increasingly recognized but still underexplored aspect of laryngeal cancer survivorship. The current literature consistently shows that issues such as communication difficulties, voice problems, swallowing issues, and other treatment-related functional limitations can significantly impact emotional health, self-esteem, social participation, and overall quality of life [16,29,46,47,48,49].

Voice problems are among the most commonly studied psychosocial aspects. Research using tools such as the Voice Handicap Index (VHI), Voice-Related Quality of Life (V-RQOL), and Speech Handicap Index (SHI) suggests that voice impairments may lead to communication avoidance, reduced social engagement, job limitations, and emotional distress [46,48,50]. Overall, the evidence indicates that successful medical treatment does not always result in full psychosocial recovery or patient satisfaction, emphasizing the need to include patient-centered outcomes in survivorship evaluations.

The literature also highlights the importance of communication reintegration and multidisciplinary rehabilitation. Comprehensive rehab programs that include speech therapy, psychosocial support, and long-term follow-up can help patients adjust after treatment and enhance patient-reported results [29]. Nonetheless, the use of rehabilitation services varied across studies, suggesting that referral processes, recognition of psychosocial needs, and access to specialized survivorship care may be inconsistent. These findings underscore the complexity of psychosocial recovery after laryngeal cancer and the necessity for personalized, multidimensional survivorship care.

### 4.3. Limited Analytical Integration Between PROMs and Objective Outcomes

Although numerous studies included both PROMs and objective functional assessments, a comprehensive analytical integration of these domains was largely absent throughout the reviewed literature. Several studies reported PROMs alongside methods such as acoustic voice analysis, videolaryngostroboscopy, swallowing evaluations, nutritional assessments, or clinician-rated functional scales. However, most of these studies analyzed outcomes separately rather than examining their relationships [17,26,27,36,51,52].

This methodological pattern was particularly evident in studies assessing voice outcomes after cordectomy, laser microsurgery, or radiotherapy. Researchers often reported objective measures like jitter, shimmer, harmonic-to-noise ratio, maximum phonation time, aerodynamic analysis, or perceptual GRBAS ratings alongside voice-related PROs such as VHI or V-RQOL [17,27,52]. However, few studies have examined whether objective acoustic impairments are linked to perceived communication difficulties or overall quality of life. Several researchers have noted that objective vocal performance often does not align with patients’ perceptions, suggesting that acceptable acoustic results can coexist with significant self-reported communication challenges [17,26,52].

A similar discrepancy was noted in swallowing assessments. Peretti et al. showed that MDADI scores did not significantly differ across treatment groups, even though videofluoroscopic and endoscopic evaluations revealed measurable differences [27]. Similarly, Olthoff et al. highlighted that subjective adaptation mechanisms might partially compensate for ongoing dysfunction, possibly concealing clinically relevant impairments when only PROMs are used [51]. These results suggest that patient-reported adaptation and actual physiological recovery may not progress together.

Few studies have used comprehensive multidimensional assessment models that incorporate functional, nutritional, and quality-of-life outcomes. For example, Zhu et al. combined videofluoroscopic swallowing evaluations, nutritional assessments, and quality-of-life questionnaires to evaluate the impact of swallowing rehabilitation following laryngeal cancer treatment [36]. Their findings showed improvements across swallowing function, nutritional status, and overall quality of life, highlighting the potential of integrated survivorship models. However, even in such studies, there was a lack of predictive modeling and multivariable analysis to explore the interactions between objective impairments and patient perceptions.

This limitation makes interpretation harder, limits personalized rehab planning, and complicates understanding how physiological impairments relate to patient experience.

### 4.4. Future Directions and Clinical Implications

This review underscores the importance of establishing more standardized and multidimensional frameworks for survivorship assessment in laryngeal cancer research. Future research should focus on creating core outcome sets that encompass voice, swallowing, nutritional, psychosocial, and supportive care aspects, enhancing comparability across studies and capturing the full complexity of recovery after treatment. Improved integration of PROMs with objective functional assessments—such as acoustic voice analysis, swallowing tests, and nutritional evaluations—could deepen our understanding of how physiological impairments relate to patient perceptions. Additionally, multicenter prospective longitudinal studies employing standardized assessment protocols are essential to boost external validity and inform predictive models for rehabilitation. Fully integrating PROMs into routine survivorship care could lead to earlier detection of unmet functional and psychosocial needs, supporting more patient-centered approaches to rehabilitation following laryngeal cancer treatment.

Emerging approaches such as transoral robotic surgery (TORS) are attracting increasing attention for their potential to improve functional recovery in the treatment of specific laryngeal cancers, especially supraglottic ones. Recent research has focused on outcomes such as swallowing, airway management, and recovery after TORS [53]. As more studies are published, future research should include standardized PROM assessments to better understand patient perspectives and enable comparisons between different treatment options.

An additional way to improve survivorship assessment is through the growing use of electronic PROMs (ePROMs) and digital monitoring platforms. These tools allow for real-time collection of patient-reported outcomes, ongoing symptom tracking, automatic alerts for significant health changes, and integration with electronic health records. In laryngeal cancer survivorship, digital platforms can support more thorough monitoring of voice, swallowing, nutrition, psychosocial health, and supportive care needs while minimizing the burden of traditional paper assessments. Additionally, ePROM systems can more effectively integrate patient-reported and clinical data, enabling more tailored rehabilitation and long-term follow-up. Future studies should investigate how digital outcome tracking can enhance the use of PROMs in laryngeal cancer management.

### 4.5. Strengths and Limitations

This review has notable strengths. It offers a thorough mapping of current literature on PROM use in laryngeal cancer survivorship, including many studies across various treatment methods and functional areas. It also provides a multidimensional synthesis that integrates insights from voice, swallowing, nutrition, psychosocial, and supportive care. Additionally, the innovative framework examining the integration of PROMs with objective functional outcomes is a methodological contribution that can guide future research and assessment approaches in survivorship.

However, several limitations must be acknowledged. The review focused only on studies published in English, following the predefined protocol. While machine translation tools can help include studies in other languages, some relevant non-English publications might have been missed. As a result, there may be language bias, which could impact the completeness of the evidence map. As a scoping review, it did not perform a formal risk-of-bias assessment and did not base the strength of the evidence on methodological quality. The diversity in study designs, patient groups, treatments, follow-up periods, and outcome measures also prevented a quantitative analysis. An additional limitation relates to the integration framework used to classify the relationship between PROMs and objective clinical outcomes. Although developed a priori and applied consistently across the included studies, the framework was created specifically for this review and has not undergone formal validation, pilot testing, or assessment of inter-rater reliability. Consequently, it should be interpreted as a descriptive evidence-mapping tool rather than a standardized methodological instrument. Lastly, gray literature might have been underrepresented.

## 5. Conclusions

This scoping review reveals significant variability in the use of patient-reported outcome measures in laryngeal cancer survivorship research. Current assessment approaches primarily focus on voice, while areas such as swallowing, nutrition, psychosocial aspects, and supportive care are evaluated less consistently. Additionally, the literature shows limited integration between PROMs and objective functional outcomes, with most studies reporting these measures separately rather than within a comprehensive, multidimensional framework.

These results emphasize the need for standardized, comprehensive survivorship assessment models that combine subjective and objective data across various functional areas. Future longitudinal studies with harmonized assessment strategies could enhance comparability, aid personalized rehabilitation, and promote patient-centered care following laryngeal cancer treatment.

## Figures and Tables

**Table 1:** Characteristics of included studies (*n* = 95).

Characteristic	Value
Total studies	95
**Publication year**	
Median (IQR)	2016 (2012–2021)
Range	2001–2025
**Geographic distribution**	
Europe	56 (58.9%)
Asia	23 (24.2%)
North America	9 (9.5%)
South America	4 (4.2%)
Oceania	2 (2.1%)
Africa	1 (1.1%)
**Study design**	
Observational	80 (84.2%)
Interventional	15 (15.8%)
**Study design type**	
Cross-sectional	26 (27.4%)
Retrospective	21 (22.1%)
Prospective	26 (27.4%)
Randomized controlled trials	7 (7.4%)
Other designs	15 (15.7%)
**Study setting**	
Single-center	69 (72.6%)
Multicenter	26 (27.4%)
**Sample size per study**	
Median (IQR)	69 (43.5–95.5)
Range	28–2370
Total participants	10,807
**Follow-up data reported**	
Reported	79 (83.2%)
Not reported	16 (16.8%)

Data are presented as numbers (percentages), medians (interquartile ranges [IQRs]), or ranges, as appropriate. Abbreviations: IQR, interquartile range.

**Table 2:** Patient-reported outcome instruments used across included studies (*n* = 95).

PROM Instrument	*n* (%)	Category
VHI family (VHI, VHI-10, VHI-30, subscales)	43 (45.3%)	Voice
EORTC QLQ-C30	28 (29.5%)	Quality of life
EORTC QLQ-H&N35	28 (29.5%)	Head and neck–specific QoL
MDADI	11 (11.6%)	Swallowing
S-SECEL family (S-SECEL, I-SECEL)	8 (8.4%)	Psychological/communication
V-RQOL	8 (8.4%)	Voice
UW-QOL (all variants)	7 (7.4%)	Quality of life
SWAL-QOL	3 (3.2%)	Swallowing
Other instruments *	24 (25.3%)	Mixed/other

Note: Individual studies could report more than one patient-reported outcome measure (PROM); therefore, percentages do not sum to 100%. Abbreviations: EORTC QLQ-C30, European Organisation for Research and Treatment of Cancer Quality of Life Questionnaire-Core 30; EORTC QLQ-H&N35, European Organisation for Research and Treatment of Cancer Quality of Life Questionnaire–Head and Neck Module; VHI, Voice Handicap Index; MDADI, M. D. Anderson Dysphagia Inventory; SWAL-QOL, Swallowing Quality of Life Questionnaire; UW-QOL, University of Washington Quality of Life Questionnaire; S-SECEL, Self-Evaluation of Communication Experiences after Laryngectomy; QoL, quality of life. * Other instruments included SF-36, FACT-H&N, SIP, QLICP-HN, WHOQOL-BREF, HADS/HAD, PSS-HN, PSS-H&N, VoiSS, SHI, COOP/WONCA, SF-8, SF-12, DASS-21 and other less frequently reported PROMs.

**Table 3:** Use and domains of PROMs across included studies (*n* = 95).

Variable	Category	*n* (%)
PROM use		
	Used	82 (86.3%)
	Not used	11 (11.6%)
	Not reported	2 (2.1%)
Domains assessed		
	Voice	82 (86.3%)
	Psychological	69 (72.6%)
	Swallowing	62 (65.3%)
	Supportive care	39 (41.1%)
	Nutrition	21 (22.1%)

Abbreviations: PROMs, patient-reported outcome measures.

**Table 4:** Degree of integration between patient-reported outcome measures and objective clinical outcomes across included studies (*n* = 95).

Integration Level	Definition	*n* (%)
Level 0	PROMs reported without objective outcomes	33 (34.7%)
Level 1	PROMs and objective outcomes reported in parallel without statistical integration	49 (51.6%)
Level 2	PROMs statistically associated with objective outcomes (e.g., correlation, group comparison)	13 (13.7%)
Level 3	Predictive integration using multivariate models combining PROMs and objective outcomes	0 (0%)

Abbreviations: PROMs, patient-reported outcome measures.

## Data Availability

The data supporting the findings of this study are available within the article and its Supplementary Materials. Additional extracted data are available from the corresponding author upon reasonable request.

## Supplementary Materials

The following supporting information can be downloaded at https://www.mdpi.com/article/10.3390/jcm15134872/s1.

## Abbreviations

The following abbreviations are used in this manuscript:

AJCC	American Joint Committee on Cancer
EORTC QLQ-C30	European Organisation for Research and Treatment of Cancer Quality of Life Questionnaire-Core 30
EORTC QLQ-H&N35	European Organisation for Research and Treatment of Cancer Quality of Life Questionnaire–Head and Neck Module
FACT-H&N	Functional Assessment of Cancer Therapy–Head and Neck
FEES	Fiberoptic endoscopic evaluation of swallowing
GRBAS	Grade, Roughness, Breathiness, Asthenia, Strain
H&N	Head and neck
HNR	Harmonic-to-noise ratio
HRQoL	Health-related quality of life
IQR	Interquartile range
MDADI	M. D. Anderson Dysphagia Inventory
MDT	Multidisciplinary team
MeSH	Medical Subject Headings
MPT	Maximum phonation time
OR	Odds ratio
OSF	Open Science Framework
PRISMA-ScR	Preferred Reporting Items for Systematic Reviews and Meta-Analyses Extension for Scoping Reviews
PROMs	Patient-reported outcome measures
PROs	Patient-reported outcomes
QoL	Quality of life
RCT	Randomized controlled trial
RT	Radiotherapy
S-SECEL	Self-Evaluation of Communication Experiences after Laryngectomy
SD	Standard deviation
SF-36	36-Item Short Form Health Survey
SHI	Speech Handicap Index
SWAL-QOL	Swallowing Quality of Life Questionnaire
TLM	Transoral laser microsurgery
UW-QOL	University of Washington Quality of Life Questionnaire
VEES	Videoendoscopic evaluation of swallowing
VFSE	Videofluoroscopic swallowing evaluation
VFS	Videofluoroscopic swallowing study
VHI	Voice Handicap Index
VHI-10	Voice Handicap Index-10
VHI-30	Voice Handicap Index-30
V-RQOL	Voice-Related Quality of Life questionnaire
WHOQOL-BREF	World Health Organization Quality of Life-BREF
