# Supplementary material for: Patient-Reported Outcomes and Functional Recovery After Treatment for Laryngeal Cancer: A Scoping Review of Instruments, Domains, and Clinical Integration
Source: J Clin Med. 2026 Jun 23;15(13):4872. doi: 10.3390/jcm15134872 (PMC13362266; doi:10.3390/jcm15134872)
Supplement: Supplementary file 1 [file jcm-15-04872-s001.zip › Supplementary File S1.pdf]

PubMed/MEDLINE search strategy

("laryngeal cancer"[MeSH Terms] OR "laryngeal cancer" OR "larynx cancer" OR "glottic cancer" OR "supraglottic cancer")
AND
("patient-reported outcome" OR "patient reported outcome" OR "PROM" OR "PROMs" OR "quality of life" OR "QoL" OR "health-related quality of life" OR "HRQoL")
AND
("voice" OR "voice outcome" OR "voice quality" OR "speech" OR "swallowing" OR "dysphagia" OR "functional outcome" OR "functional recovery")

Scopus search strategy

(TITLE-ABS-KEY("laryngeal cancer" OR "larynx cancer" OR "glottic cancer" OR "supraglottic cancer"))
AND
(TITLE-ABS-KEY("patient-reported outcome" OR "PROM" OR "PROMs" OR "quality of life" OR "QoL" OR "HRQoL"))
AND
(TITLE-ABS-KEY("voice" OR "speech" OR "swallowing" OR "dysphagia" OR "functional outcome" OR "functional recovery"))

Web of Science search strategy

TS=("laryngeal cancer" OR "larynx cancer" OR "glottic cancer" OR "supraglottic cancer")

AND

TS=("patient-reported outcome" OR "patient reported outcome" OR "PROM" OR "PROMs" OR "quality of life" OR "QoL" OR "health-related quality of life" OR "HRQoL")

AND

TS=("voice" OR "voice outcome" OR "voice quality" OR "speech" OR "swallowing" OR "dysphagia" OR "functional outcome" OR "functional recovery")
